# Starmaya: The First Arabica F1 Coffee Hybrid Produced Using Genetic Male Sterility

**DOI:** 10.3389/fpls.2019.01344

**Published:** 2019-10-22

**Authors:** Frédéric Georget, Lison Marie, Edgardo Alpizar, Philippe Courtel, Mélanie Bordeaux, Jose Martin Hidalgo, Pierre Marraccini, Jean-christophe Breitler, Eveline Déchamp, Clément Poncon, Hervé Etienne, Benoit Bertrand

**Affiliations:** ^1^CIRAD, UMR IPME, Montpellier, France; ^2^IPME, Université de Montpellier, IRD, CIRAD, Montpellier, France; ^3^Plant material, ECOM, Exportadora Atlantic, Managua, Nicaragua; ^4^FONDATION NICAFRANCE, Managua, Nicaragua

**Keywords:** Arabica coffee, F1 hybrid seed, male sterility, micropropagation, commercial production

## Abstract

In the present paper, we evaluated the implementation of a seed production system based on the exploitation of male sterility on coffee. We studied specifically the combination between CIR-SM01 and Marsellesa^®^ (a Sarchimor line), which provides a hybrid population called Starmaya. We demonstrated that the establishment of seed garden under natural pollination is possible and produces a sufficient amount of hybrid seeds to be multiplied efficiently and economically. As expected for F1 hybrid, the performances of Starmaya are highly superior to conventional cultivars. However, we observed some heterogeneity on Starmaya cultivar in the field. We confirmed by genetic marker analysis that the off-types were partly related to the heterozygosity of the CIR-SM01 clone and could not be modified. Regarding the level of rust resistance of Starmaya cv., we saw that it could be improved if Marsellesa was more fully fixed genetically. If so, we should be able to decrease significantly the percentage of rust incidence of Starmaya from 15 to 5%, which would be quite acceptable at a commercial level. Starmaya represents the proof of concept for the mass propagation of Arabica F1 hybrid seeds using male sterility. Finally, we discuss the possibility to increase the number of hybrid varieties produced by seed, exploring some initiatives to identify male sterility markers to induce male sterility on any conventional cultivar. This would definitively open up the universe of known Arabica cultivars to be used in breeding new F1 hybrids.

## Introduction

Due to self-fertilization of the allotetraploid *Coffea arabica* (2n = 4X = 44) species, Arabica cultivars are traditionally disseminated by seeds. This is not possible for F1 Arabica hybrids, as they are highly heterozygous and their characteristics segregate in F2 progeny produced by self-pollination. The advantages of using Arabica hybrids were first demonstrated in Kenya with the complex hybrid cultivar Ruiru 11, developed in the 1970s and released in 1986 to be cultivated in Kenya (Walyaro 1983; Van der Vossen, 1985), then in Tanzania (Teri et al., 2005; Van der Vossen et al., 2015). Breeding strategies aiming to develop new F1 hybrid cultivars (from two parents) of Arabica coffee were proposed simultaneously in Ethiopia (Bellachew, 1997) and in Central America (Bertrand et al., 1997; Bertrand et al., 2011). In Central America, the strategy was based on crossing American cultivars with wild Ethiopian accessions. Like many other hybrid plants, *C. arabica* F1 hybrids possess genetic and agronomic advantages (Gallais, 2009), such as higher and more stable yields, more vigor, disease resistance, better cup quality, and adaptability to agroforestry systems (Bertrand et al., 2005; Bertrand et al., 2011). These authors showed that F1 hybrids produced 11–47% higher yield than the best cultivars along with significantly higher or identical 100-bean weight and performed identically for fertility. To assess whether using hybrids represents substantial genetic progress on terms of productivity and full-sun cropping systems, the new F1 hybrids were grown in the same conditions as the best American cultivars. The results showed that yields of hybrids were earlier and superior to those of American cultivars. The hybrids were also more stable than the American cultivars in all environments tested. In the agroforestry system, the mean yield of hybrids was 58% higher than that of American cultivars, while the mean yield of hybrids in the full-sun system was 34% higher. So, by combining more productivity, better adaptability to agroforestry system, more resistance, and better cup quality, F1 hybrids are a promising alternative to conventional cultivars. Once created, several methods exist to multiply these new F1 hybrids on a large scale.

As previously reported in Kenya and Ethiopia, the first method consisted in producing F1 seeds by hand pollination (Opile and Agwanda, 1993; Bellachew, 1997). In that case, and because Arabica flowers are hermaphrodite and autogenic, it was necessary to emasculate them before pollinating by hand. For coffee, this approach has proven difficult to implement on a large scale. The second method was to produce clonal F1 hybrid plants by different micropropagation technics. In Tanzania for example, through the full participation of some 600 farmer groups, the F1 hybrids were multiplied by rooted cuttings and grafting (Magesa et al., 2013). On the other hand, the ECOM/Centre de Coopération Internationale en Recherche Agronomique Pour le Développement (CIRAD) consortium produced an average of one million F1 Arabica hybrid coffee plants per year by somatic embryogenesis (SE) in Nicaragua (Etienne et al., 2012; Etienne et al., 2018). Although this approach worked technically, it was not economically viable due to its high costs linked to manpower and the infrastructures required (laboratories and nurseries) (Van der Vossen et al., 2015). We recently showed that the cost of F1 coffee plants could be reduced by the half, if plants were produced by a combination of SE and rooted mini-cuttings technics (Georget et al., 2015b). A 6-month-old nursery hybrid plant propagated from a somatic seedling and ready for the field, which has a production cost of around 0.60 USD, whereas the same hybrid plant produced by rooted mini-cuttings has a production cost of around 0.27 USD. However, the price of such F1 hybrids produced first by SE, then re-multiplied by rooted minicuttings, is still 1.5 times the cost of traditional seedling, which still remains too high and constitutes a major deterrent for small coffee farmers (Georget et al., 2017).

Lastly, the third method consisted in producing F1 seeds in seed gardens using a sterile male line as the female plant (Georget et al., 2015a). Male sterility is common in flowering plants, but its application in hybrid breeding and seed production is limited because of the inability to propagate a pure sterile male line for commercial hybrid seed production (Chang et al., 2016). There is therefore a limited number of publications reporting on the use of sterile male genotypes to produce hybrid seeds at commercial level. Numerous hybrid crops that are significant components of world production (>50%) such as tomato and maize are produced by hand emasculation, while spinach and the cucurbits hybrid seeds are produced by monoecy and the brassicas by self-incompatibility (Havey, 2004). For other crops, such as rice, wheat, and sorghum, commercial quantities of hybrid seeds can only be obtained by using sterile male female parents created by chemical or genetic manipulations (Wu et al., 2016). In this case, the successful application of such systems for large-scale hybrid seed production depends on whether the sterile male female parent line can be multiplied efficiently and economically (Perez-Prat and Van Lookeren Campagne, 2002). Compared with other methods, these hybrid seeds offer the advantages of being produced in quantity, easily handled, stored, and transported, and are much more affordable for farmers. With an annual renewal rate estimated at over one billion trees for *C. arabica* (B. Bertrand, personal communication), this type of propagation by seeds should allow rapid and large-scale dissemination of Arabica F1 hybrids.

In this paper, we evaluated the implementation of a seed production system using the *C. arabica* sterile male CIR-SM01, and we evaluated the performance of the first F1 hybrid, “Starmaya,” obtained by male sterility in comparison with conventional cultivars.

## Materials and Methods

### *C. arabica* Sterile Male CIR-SM01

**Figure 1:**
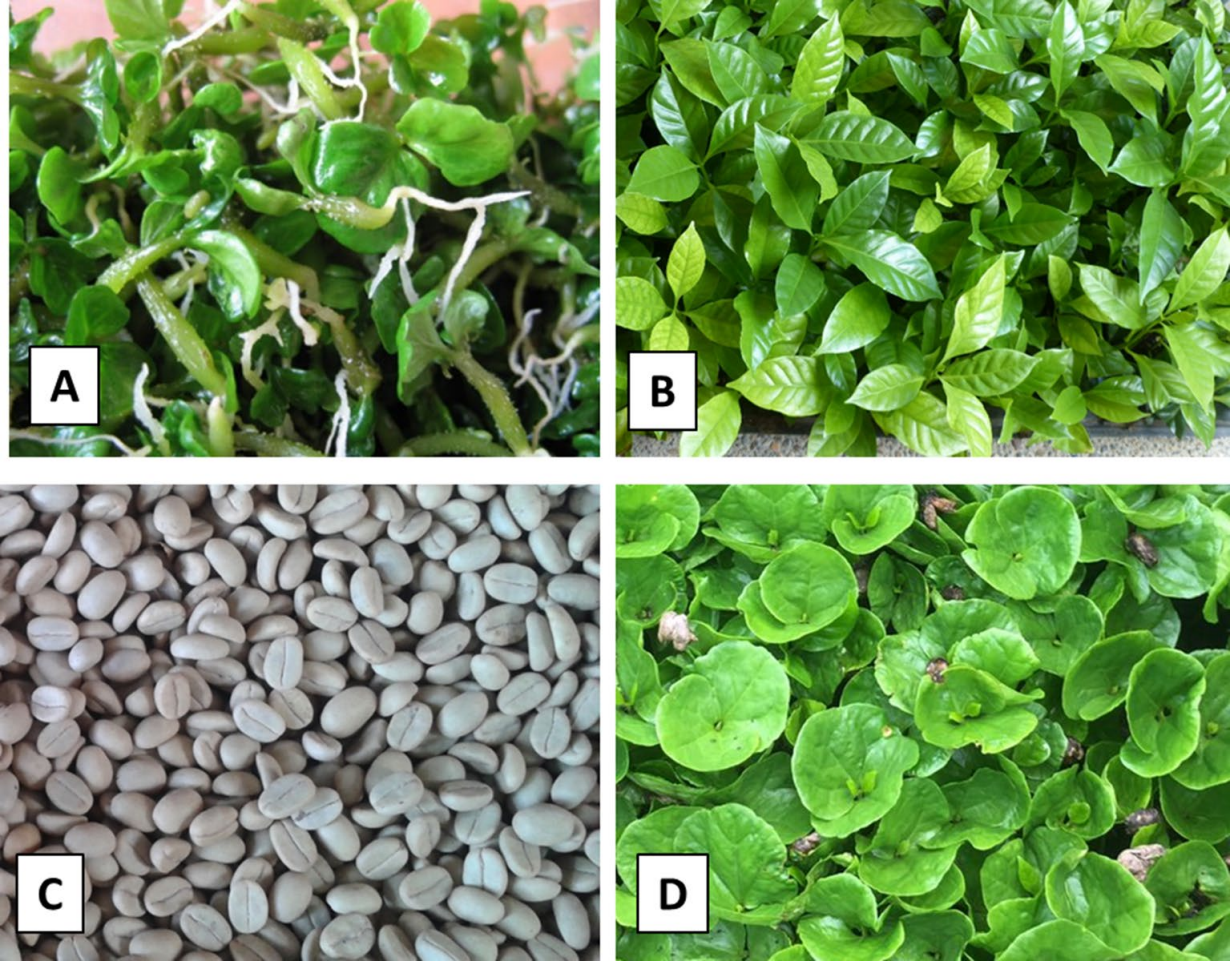
Production of Starmaya F1 *C. arabica* hybrid. **(A)** Pre-germinated embryos of sterile-male CIR-SM01 produced by somatic embryogenesis. **(B)** Plantlets of sterile-male CIR-SM01 derived from somatic embryos and acclimatized in trays during 4 months. **(C)** Seeds of *C. arabica* Marsellesa^®^ variety. **(D)** 2-month-old plants of Marsellesa^®^ variety obtained after seed germination.

In 2001, researchers from CIRAD found a natural tall growth habit mutant (called CIR-SM01) of *C. arabica* without any pollen production, by screening Ethiopian plants at the Centro Agronómico Tropical de Investigación y Enseñanza (CATIE) germplasm bank for coffee in Costa Rica. Plants of CIR-SM01 were then multiplied *in vitro* in clonal form by SE (Atlantic S.A. laboratory, Sebacco, Nicaragua). Somatic embryos derived from plantlets were first acclimatized in plastic tunnels for 2 months in trays, then hardened in nurseries for 4 months before planting in the field and further used as mother receptors (**Figures 1A, B**). After this process, it was checked that the sterile male phenotype was not affected by temperature or photoperiod-sensitive, as described in rice for example (Zhang et al., 1994). This mutation displayed complete and stable sterility, whatever the environmental conditions and the age of the plant (juvenile or mature). The molecular basis of this mutation is still unknown, but we found in a previous study (Dufour et al., 1997) that phenotypic expression of this male sterility is functional, occurring during microsporogenesis, and is the product of a malfunction of the tapetum in the early microspore stages. At the same time, we planted four F2 populations (1,250 plants), and we checked that the mutation was recessive, giving a proportion of 18.3% sterile male plants. This proportion may have been the product of a Mendelian recessive epistatic interaction with a 9:4:3 ratio.

### Marsellesa® Cultivar of *C. arabica*

The Marsellesa^®^ cultivar (https://www.upov.int/pluto/en/) is a Sarchimor cultivar selected (F8) with complete resistance to leaf rust disease (coffee leaf rust caused by *Hemileia vastatrix*) and to coffee berry disease (caused by *Colletotrichum kahawae*), as described in the World Coffee Research variety catalog (https://varieties.worldcoffeeresearch.org/). Compared with a traditional dwarf cultivar, such as Caturra red, Marsellesa^®^ produces up to 10% more, with good organoleptic qualities. Seeds from Marsellesa^®^ were taken from a plantation located on the “La Cumplida” farm (Matagalpa, Nicaragua) and sprouted for 45 days in a germinator (**Figures 1C, D**). The resulting seedlings were transferred to nursery bags for 4 months to be fully acclimatized, then planted in the field to be further used as pollen donors.

### Creation of the Starmaya Hybrid Cultivar

In a preliminary study, we first investigated the possibility of producing F1 hybrid seeds by crossing (by hand pollination) the sterile male CIR-SM01 separately with several *C. Arabica* cultivars with a dwarf growth habit (e.g., Caturra red, Catuai 44, IAPAR59, and Marsellesa^®^) used as pollen donors and evaluating the agronomic performances of the corresponding F1 progenies under field growing conditions (data not shown). In this trial, all plants were fertile with better vegetative vigor than the control (Caturra red). Of the four combinations tested, the best cross was the “CIR-SM01 × Marsellesa^®^” F1 population, which we called “Starmaya.” This candidate cultivar yielded 30% more of green beans than Marsellesa^®^, with a standard to good beverage quality and rust resistance. The combinations with Caturra red and Catuai 44 were discarded due to high susceptibility to rust, and the combination with IAPAR59 presented a cup quality inferior to the Caturra red standard.

This first step was carried out on a few plants, so we decided to evaluate the agronomic performance of this new cultivar on a larger scale by comparing it with conventional ones and to validate the concept of commercially producing Starmaya hybrid seeds using male sterility and natural pollination.

### Set up of the Starmaya Seed Garden

**Figure 2:**
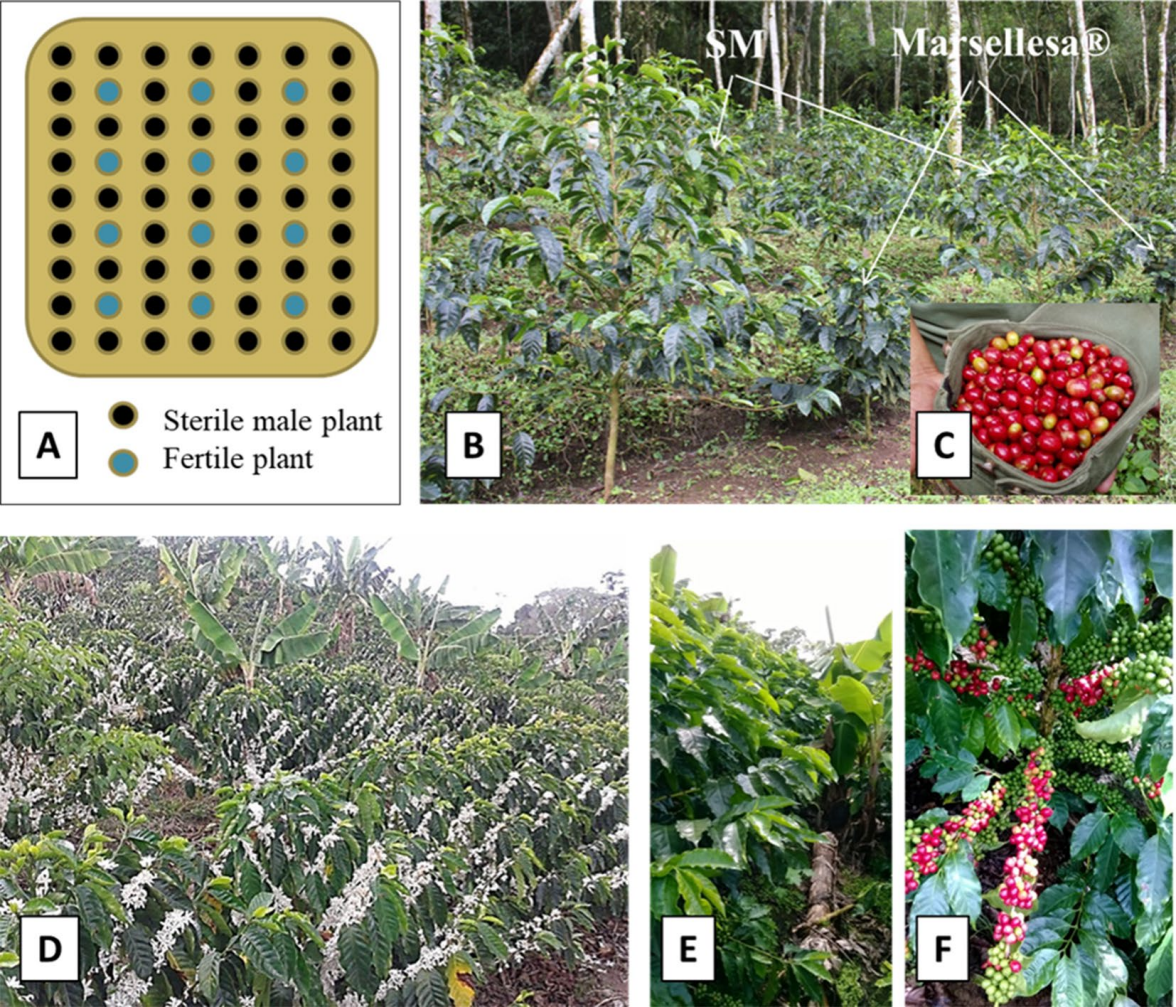
Experimental seed garden used to produce seeds of Starmaya F1 hybrid of *C. arabica*. **(A)** Scheme of planting in seed garden to produce seeds of Starmaya F1 hybrid. The fertile plants (in blue) producing pollen correspond to Marsellesa^®^ pure line variety. The sterile male plants corresponding to CIR-SM01 produce Starmaya seeds. In seed garden, the ratio is approximately one plant of Marsellesa for 4 plants of sterile male (SM) CIR-SM01. **(B)** Seed garden established in the field of “La Cumplida” farm located in Matagalpa (Nicaragua). **(C)** First seeds of Starmaya F1 hybrid harvested in seed garden in 2011. **(D)** First flowering (in 2014) of Starmaya F1 hybrid plots established at “La Cumplida” farm. **(E)** Starmaya plants in 2015. **(F)** Aspect of producing branches of Starmaya producing the first commercial yield in 2015.

To produce hybrid seeds, we planted CIR-SM01 together with Marsellesa^®^, assuming that the pollen of Marsellesa^®^ would be able to pollinate the sterile male naturally. The proportion was approximately one pollen donor for four sterile male plants used as mothers. The first experimental seed garden, isolated from alien pollen contamination, was planted in 2009 on the “La Cumplida” farm (950 m above sea level, district of Matagalpa, Nicaragua) on half a hectare (2,000 trees), with 1,600 CIR-SM01 somatic seedling plants and 400 Marsellesa^®^ plants as pollen donors. The planting density was 1.25 m along the row and 2 m between rows, i.e., 4,000 plants per hectare (**Figure 2**).

### Multi-Site Trial to Assess the Performances of the Starmaya F1 Hybrid

A multi-site trial was conducted across seven environments in Nicaragua for 5 years (2013 to 2018). The objective was to test the Starmaya F1 hybrid in multiple environments, in order to compare it with two conventional cultivars, Caturra red and Marsellesa^®^. The agronomic management plan was identical at each site. The trials were established at seven sites ranging from 710 to 1,250 m above sea level. Implementation was identical for each site. Each site had five blocks, and each cultivar plot comprised 20 plants of each genotype. The Starmaya plot and the two control plots were randomly distributed in each block. The spacing between rows was 2.0 m, and the spacing between trees along a row was 1.5 m, corresponding to a planting density of around 3,300 trees per hectare. The sites are described in **Table 1**.

**Table 1:** List of parcel locations where the hybrid Starmaya cultivar was tested in multi-environment trials in Nicaragua.

Location	Department	Name of plot	Elevation (m.a.s.l.)*
Las Colinas	Boaco	San Jorge	710
Las Joyas	Jinotega	La Ceiba	715
La cueva del Tigre	Matagalpa	La Laguna	850
Zaragoza	Jinotega	El Guabal	1030
Las Marias	Nueva Segovia	Lote Siete	1190
La Aurora	Matagalpa	Los Cipes	1240
Albania	Matagalpa	Validacion	1250

*m.a.s.l., meters above sea level.

Productivity was recorded over 3 years for each variety plot. The growth parameters were measured for five plants per genotype per block on 2-year-old plants. Rust resistance was evaluated for 10 plants per genotype per block in 2017 and 2018.

Rust was evaluated on a scale of 0 to 4, with 0 = no symptoms, 1 = some rust spots without sporulation, 2 = majority of leaves with rust spots without sporulation, 3 = majority of leaves with rust sporulation, and 4 = majority of leaves with rust sporulation and with defoliation. For plant morphology, the volume was estimated by the formula LP × H, where LP = length of the two longest primary branches (centimeters) and H = the height of the tree (centimeters).

For bean characteristics, we estimated the weight of 100 healthy green beans (W100), and we screened the samples (5 kg for each genotype) through sieves (S14 to S20). Cup quality was assessed by six expert testers from Illy Caffè, with eight descriptors (aroma, flavor, aftertaste, acidity, body, balance, overall, and defects). A final score was assigned to each sample according to the Specialty Coffee Association (SCA) protocol (http://www.scaa.org/?page=resources&d=cupping-standards). 

To estimate the stability of Starmaya across the range of environments, productivity data were analyzed following the additive main effects and multiplicative interaction (AMMI) model. This model takes into account genotype and environment interactions (Purchase et al., 2000; Bose et al., 2014; Darai et al., 2017), with the Agricolae package in R. AMMI analyses were used to partition genotype × environment deviations into different interaction principal component axes (IPCA). AMMI analysis first fits additive effects for host genotypes and environments by the usual additive analysis of variance procedure and then fits multiplicative effects for G × E (genotype × environment) by a principal component analysis (PCA).

AMMI Model:

$$Yij=\mu +gi+ej+\sum_{k=1}^{n}\lambda k\;\alpha ik\;\gamma jk+eij$$

where µ is the general mean. Y_ij_ is the yield of the i_th_ genotype in the j_th_ environment. g_i_ is the i_th_ genotype mean deviation. e_j_ is the j_th_ environment mean deviation. λ_k_ is the square root of the eigenvalue of the PCA axis k, α_ik_ and γ_jk_ are the principal component scores for PCA axis k of the i_th_ genotype and the j_th_ environment, respectively. ε_ij_ is the residual (Zobel, 1998).

The AMMI stability value (ASV) described by Purchase et al. (2000) was calculated as follows:

$$ASV=\sqrt{{\left[\frac{IPCA1\;sum\;of\;square}{IPCA2\;sum\;of\;square}(IPCA1\;score)\right]}^{2}+{\left(IPCA2\;score\right)}^{2}}$$

where SSIPCA1/SSIPCA2 is the weight given to the IPCA1 value by dividing the interaction principal component analysis axis I (IPCA1) sum of squares by the IPCA2 sum of squares. The higher the IPCA score, either negative or positive, the more specifically adapted a genotype is to certain environments. Lower ASV scores indicate a more stable genotype across environments.

### Setup of a Starmaya Commercial Field

F1 seeds of Starmaya and Marsellesa^®^ were planted at La Cumplida (2012) in a field of 8 ha (1.25 m along rows × 2.25 m between rows). The 8 ha were divided into four blocks of 2 ha. Each block was subdivided in two equal plots of 1 ha for each genotype. We obtained a little production in 2014, but the first commercial production was in 2015. Four years after planting (2016), plant height, stem diameter, internode number, primary branch mortality, and primary branch length were measured on 300 plants randomly chosen for each genotype in the four blocks. Yield was measured as the quantity of green beans produced for each plot and expressed in bags of 46 kg ha^-1^.

Resistance to leaf rust was also evaluated by analyzing 300 plants of both varieties randomly chosen in the four previously described blocks. Rust resistance incidence was assessed during the rainy season, considered as the peak of fungus infestation observed on the farm on susceptible cultivars (i.e., Caturra red cv.). A plant was considered as susceptible if sporulation zones were observed on the abaxial surfaces of the leaves. The color of young leaves and shape of the plants (dwarf or tall) were observed three times and repeated if necessary when the information was ambiguous, until a consensus was reached. Marsellesa^®^ is considered completely resistant to the main races prevailing in Nicaragua, while CIR-MS01 is susceptible to this fungus.

The coffee beans of Marsellesa^®^ and Starmaya were classified according to their defects, size distribution (sieve S13 to S20), and sensorial analyses. After the 2015–2016 harvest, 100 kg of cherries of each variety was processed by the wet method and was dried in the sun to approximately 11% moisture. The beans were then passed through a series of sieves, thereby being divided into classes of small (sieves 13 and 14), medium (15 to 17), and large (sieves 19 and 20) beans. The percentages of defects and exportable parchment coffee were evaluated for each cultivar following procedures routinely used by Exportadora Atlantic S.A factories at Sebaco in Nicaragua (ECOM group).

Sensorial analyses of Marsellesa^®^ and Starmaya were performed twice with two trained tasters, based on the SCA protocol and using scales from 0 to 10 for the evaluated attributes. The Starmaya and Marsellesa^®^ cultivars where then compared with Caturra red cultivar harvested from a commercial plot located close to the experiment (200 m). The data corresponded to the mean of the two sensorial evaluations.

#### Simple Sequence Repeat Analysis

In order to identify the homozygous/heterozygous allele patterns, as well as any existence of alien alleles, DNA was extracted from the leaves of 10 plants randomly selected for both parents (♂ Marsellesa^®^ and ♀CIR-SM01) and Starmaya F1 hybrids for further simple sequence repeat (SSR) analysis. In this case, leaf samples of CIR-SM01 were taken from plants grown in the seed garden and those of Starmaya and Marsellesa^®^ from plants grown in the agronomic trial. The SSR primer pairs used corresponded to those previously reported by Anthony et al. (2002) and Gichuru et al. (2008) (**Table 2**).

**Table 2:** List of primers used in simple sequence repeat analysis. The primer sequences, the range size (in base pairs) of amplified fragment, and annealing temperatures used in polymerase chain reaction reactions are indicated for each primer pair.

Primer name	Oligo name		Sequence	Fragment size range (bp)	Annealing temperature
Sat 11	CO220	F	ACCCGAAAGAAAGAACCAA	143–145	35
		R	CCACACAACTCTCCTCATTC		
Sat 225	CO221	F	CATGCCATCATCAATTCCAT	265–298	35
		R	TTACTGCTCATCATTCCGCA		
Sat 24	CO223	F	GGCTCGAGATATCTGTTTAG	148–172	35
		R	TTTAATGGGCATAGGGTCC		
Sat 32	CO227	F	AACTCTCCATTCCCGCATTC	105–133	35
		R	CTGGGTTTTCTGTGTTCTCG		
Sat 47	CO229	F	TGATGGACAGGAGTTGATGG	117–150	35
		R	TGCCAATCTACCTACCCCTT		
Sat 235	CO222	F	TCGTTCTGTCATTAAATCGTCAA	227–261	35
		R	GCAAATCATGAAAATAGTTGGTG		
Sat 254	CO225	F	ATGTTCTTCGCTTCGCTAAC	202–220	35
		R	AAGTGTGGGAGTGTCTGCAT		
Sat 29	CO226	F	GACCATTACATTTCACACAC	119–135	35
		R	GCATTTTGTTGCACACTGTA		
Sat 244	CO303	F	GCATGTGCTTTTTGATGTCGT	278–308	35
		R	GCATACTAAGGAATTATCTGACTGCT		
CBD-Sat 207	CO308	F	GAAGCCGTTTCAAGCC	83–93	35
		R	CAATCTCTTTCCGATGCTCT		

The polymerase chain reaction (PCR) reactions were performed in a 15-μl final volume comprising 30 ng of genomic DNA, 2 µmol ml^-1^ of magnesium dichloride, 300 nmol ml^-1^ of deoxyribonucleotide triphosphates, and 7.5 μl of 2× PCR buffer (Type it, Qiagen), 1.0 μM each of forward and reverse primer (10 µM). Amplifications were carried out in a thermal cycler (Eppendorf) programmed at 94°C for 5 min (initial denaturation), followed by 94°C for 30 s, annealing temperature (depending on each pair) for 30 s, and 72°C for 1 min (extension) for 35 cycles, followed by a final extension step at 72°C for 5 min. PCR samples were then run on a capillary electrophoresis 3130XL with an internal standard (homemade). The SSR analysis was provided by the ADNid company (http://www.adnid.fr, Montpellier, France).

## Results and Discussion

### Potential Starmaya F1 Hybrid Seed Production of the Seed Garden

Based on the results obtained from the CIR-SM01 and Marsellesa^®^ seed garden field, we found that natural pollination was efficient for producing Starmaya F1 hybrid seeds. In terms of seed production, an average of 100–150 kg of F1 hybrid seeds (data not shown) was harvested per year from this experiment (0.5 ha). These quantities amounted to potential production of approximately half a million hybrid seeds per hectare of seed garden, which should enable the planting of over 100–120 ha of Starmaya F1 hybrid coffee each year. The sterile male CIR-SM01 produced poorly compared with commercial cultivars, which usually produce around two million seeds under similar agronomic conditions.

The cost of the seed produced by this sterile male progenitor was estimated to be about 0.07 USD per unit, corresponding to half the price of a coffee hybrid plantlet propagated by SE (Georget et al., 2017). This difference is mainly due to the fact that seed gardens are less expensive to set up and maintain than *in vitro* clonal propagation laboratories. Moreover, only a few micropropagation laboratories in the world are able to commercially produce such Arabica F1 hybrids, and none of them produces more than 2 million somatic embryo-derived plantlets per year, which represents a key constraint for the democratization of using such hybrids.

No reliable statistic exists on the seed requirements for renovating coffee plantations worldwide. According to our calculations, and considering a renovation rate of 2 to 5% per year for Arabica crops estimated to cover 7 million hectares, it would be necessary to produce 250 to 500 million trees annually. Of course, this greatly depends on the coffee prices of international markets. Under a high coffee price, more than 1 billion plants could be required yearly for renovation worldwide. However, this requirement should not exceed 200–400 million plants per year when coffee prices are low. Consequently, 50 to 100 million plants of *C. arabica* F1 hybrids should be produced per year to have a major impact with the dissemination of such hybrids at the international level (Bertrand B., personal communication). In that case, this could be achieved by using 100 to 200 ha of seed garden with a sterile male coffee accession such as CIR-SM01. According to our current knowledge, it would be impossible to produce the same amount of F1 hybrids by SE.

### Starmaya Multi-Site Variety Trial

**Figure 3:**
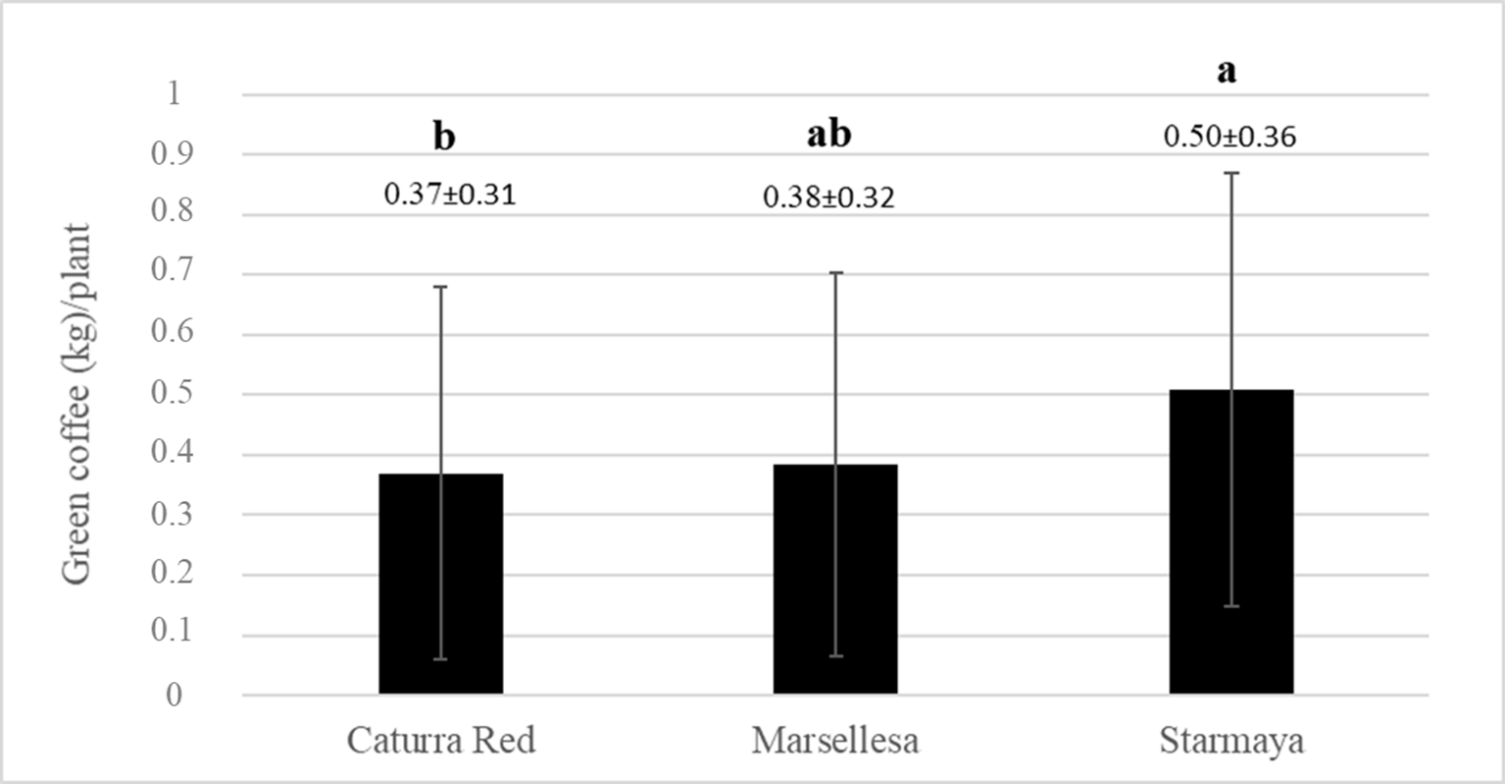
Average weight of green coffee (kilogram) per plant, for each variety. Treatment with the same letter are not significantly different at P ≤ 0.05 (Tukey’s honestly significant difference test).

**Figure 3** shows the average yields for each genotype in kilograms of green beans per plant. The graph shows that the Starmaya hybrid produced more than traditional Caturra red and Marsellesa^®^ cultivars. A Tukey’s honestly significant difference test was applied to determine significant differences between averages. Starmaya produced significantly more compared with Caturra red and Marsellesa^®^ (30% more per plant).

By AMMI models, we evaluated and provided a stability index, the ASV. Genotypes with a low ASV are considered stable. The ASV allowed us to identify that Starmaya was the more stable genotype having the lowest ASV value (ASV = 0.308), while Marsellesa^®^ and Caturra red, with an ASV of 0.807 and 0.819, respectively, appeared less stable.

**Figure 4:**
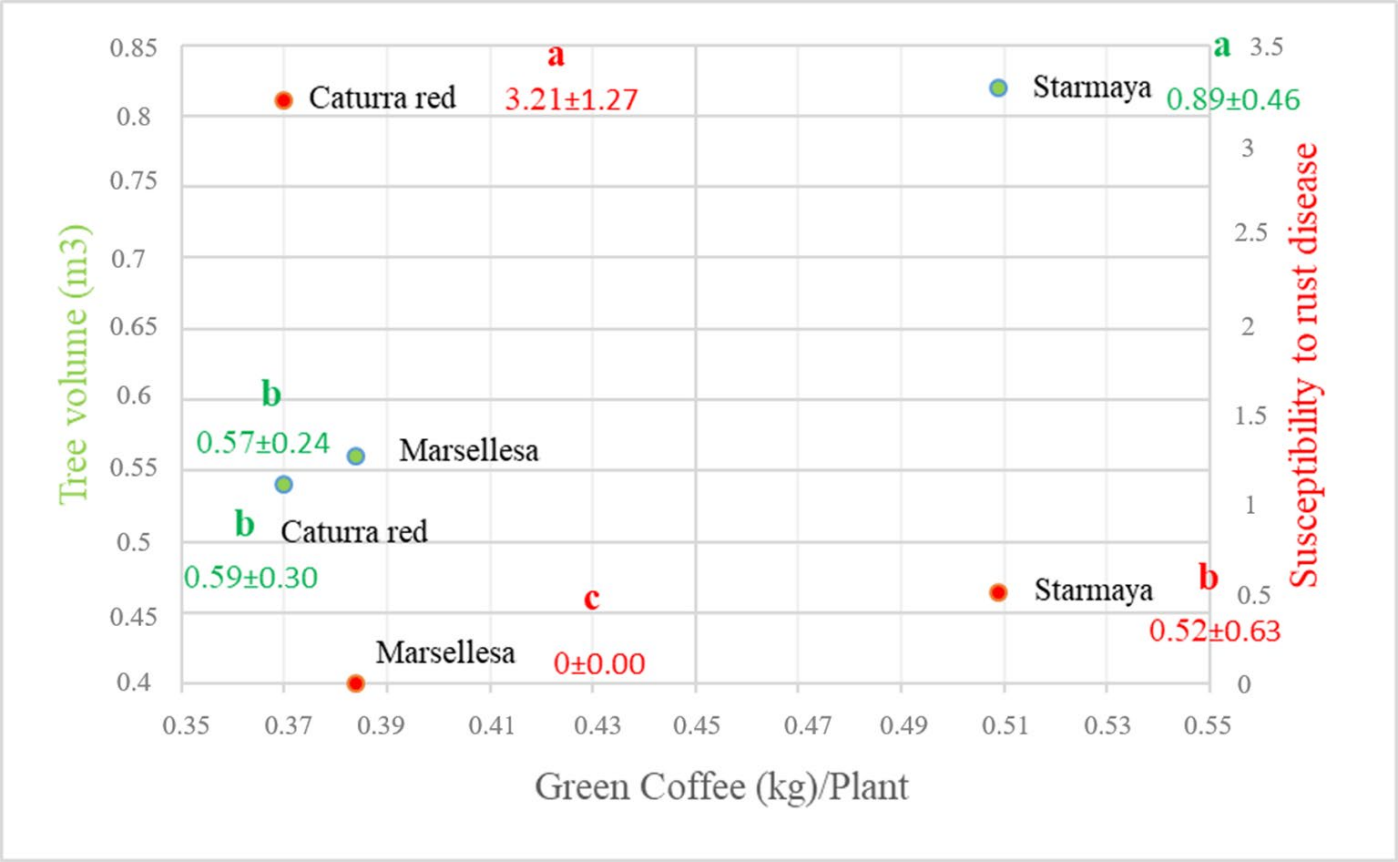
Relationship between productivity and two parameters: tree volume and susceptibility to rust. Treatment with the same letter are not significantly different at P ≤ 0.05 (Tukey’s honestly significant difference test).

The tree volume of Starmaya (**Figure 4**) (0.89 m^3^) was greater than that of Marsellesa^®^ and Caturra red (0.59 m^3^). The differences are mainly due to lateral length and reflect strong vegetative vigor. Starmaya was found to be less susceptible to rust than Caturra red, but more than Marsellesa^®^, due to the presence of some susceptible plants. As rust resistance background in Marsellesa^®^ is conferred by dominant genes, we concluded that some Starmaya plants do not inherit rust resistance.

**Figure 5:**
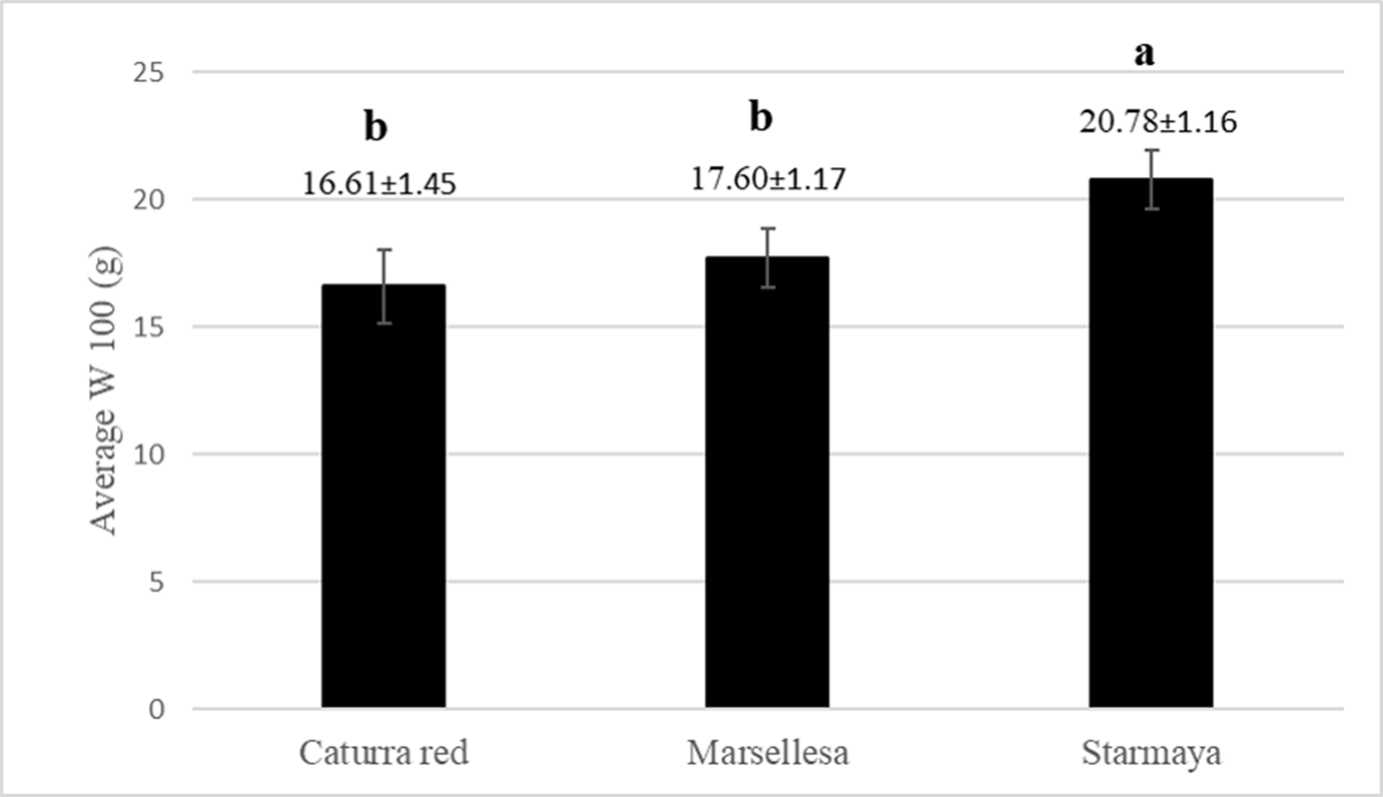
Average weight (gram) of 100 healthy green beans per variety (W100). Treatment with the same letter are not significantly different at P ≤ 0.05 (Tukey’s honestly significant difference test).

The W100 appeared higher for the Starmaya hybrid compared with the conventional cultivars (**Figure 5**). Starmaya had an average weight of 100 healthy green beans 25% higher than Caturra red. The higher W100 weight indicated better filling during bean development.

**Figure 6:**
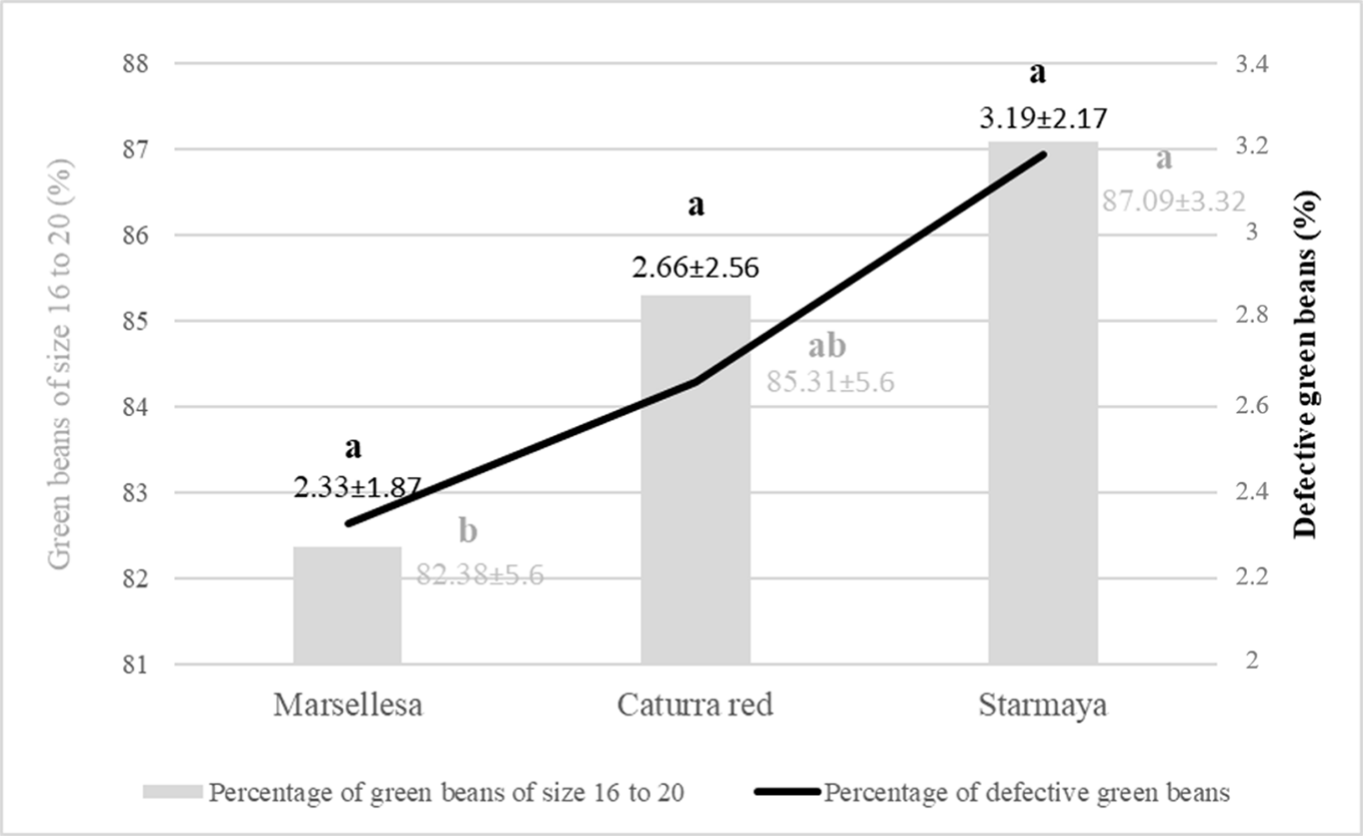
Percentage of green beans of size 16 to 20 and defective green beans by variety. Treatment with the same letter are not significantly different at P ≤ 0.05 (Tukey’s honestly significant difference test).

Bean grade also plays an important role in marketing. The purchase price of bean is related to the density and size of the bean, and its healthy physical appearance. Beans are classified in 64th of an inch increments, with sizes between 14 to >19. Beans with screen sizes up to 15 are considered the highest quality in terms of size. **Figure 6** shows, for each cultivar, the percentage of beans of size 16 to 20 and the percentage of defective beans. Starmaya gave a higher percentage of green beans of size 16 to 20 than the two controls and 1% more defective beans.

As concerns sensorial perception, according to the SCA method, coffees with a score of less than 80 are considered to be of inferior quality. Coffee beverages that have a score between 80 and 84.99 are very good, and beyond 85, they are excellent or exceptional. Cup quality varies depending on genotypes and altitudes. **Table 3** shows that Starmaya was better rated than Caturra red and obtained scores over or equal to 80, being very good at the highest altitude (>83).

**Table 3:** Score of sensorial perception according to the Specialty Coffee Association method and made by Illy Caffè (Italy).

Genotypes/Elevation	710 m.a.s.l*	1,190 m.a.s.l	1,240 m.a.s.l	1,250 m.a.s.l
Caturra red	72.5	77.7	78.9	74.4
Starmaya	79.2	80	80	83.1

* m.a.s.l, meters above sea level.

### Phenotypic and Yield Evaluation of Starmaya Under Agronomic Conditions

The phenotypic characteristics of Marsellesa^®^ and Starmaya were evaluated in year 4 and are reported in **Table 4**. For all the phenotypic parameters studied (except the number of primary branch internodes at level 15 from the apex), the comparison showed that Starmaya plants were more vigorous than those of Marsellesa^®^. For example, Starmaya plants were always significantly taller and had a larger collar diameter than Marsellesa^®^. Moreover, Starmaya plants also had longer primary branches and larger internodes than those of Marsellesa^®^.

**Table 4:** Phenotypic comparison of *C. arabica* Marsellesa^®^ Sarchimor cultivar and Starmaya F1 hybrid (CIR-SM01 × Marsellesa^®^).

Parameters	Marsellesa^®^	Starmaya
Stem diameter at collar (mm)	38.6 ± 4.0^b^	44.0 ± 6.1^a^
Plant height (cm)	177.5 ± 16.5^b^	206.3 ± 15.9^a^
Nb total of internodes	33.3± 3.2^b^	37.9 ± 2.7^a^
Primary branch length (cm) *level 10 from the apex* (Branch 1)	40.5 ± 7.0^b^	48.3 ± 9.6^a^
Nb of primary internode (Branch 1)	10.2 ± 1.8^b^	11.33 ± 2.1^a^
Primary branch length (cm) *level 10 from the apex* (Branch 2)	41.2 ± 7.7^b^	51.9 ± 9.0^a^
Nb of primary internode (Branch 2)	10.5 ± 1.9^b^	11.7 ± 2.1^a^
Primary branch length (cm) *level 15 from the apex* (Branch 1)	59.1 ± 6.8^b^	64.2 ± 9.3^a^
Nb of primary internode (Branch 1)	14.9 ± 1.7^a^	14.6 ± 1.7^a^
Primary branch length (cm) *level 15 from the apex* (Branch2)	58.3 ± 7.4^b^	64.0 ± 9.6^a^
Nb of primary internode (Branch 2)	13.8 ± 1.7^a^	14.6 ± 1.7^a^

Measurements were performed on 300 plants from each cultivar evaluated in 2016, 4 years after their field planting (2012). The comparison was based on an analysis of variance. Values followed by different letters are significantly different at P ≤ 0.05 (Fischer test of least significance difference).

Regarding the yield assessment, the results presented in **Table 5** show that Starmaya produced 47% more than Marsellesa^®^ in 2015–2016 (first commercial harvest) and 29% more for the second commercial harvest (2016–2017). The yield of Starmaya was still better than that of Marsellesa^®^ for the third harvest (2017–2018). After three large yields, the experimental field was pruned. Considering the average harvests, Starmaya produced up to 35% more than Marsellesa^®^ under commercial growing conditions. These results confirmed the superiority of the Starmaya F1 hybrid compared with the Marsellesa^®^ cultivar. The Starmaya F1 hybrids grown from seeds displayed similar vigor and yields compared with other F1 hybrid clones propagated by SE (Bertrand et al., 2005).

**Table 5:** Comparison of the productivity of Marsellesa^®^ and Starmaya cultivars during three harvest campaigns.

Harvest campaigns	Productivity (no. of bags of 46-kg green coffee beans per hectare)	
	Marsellesa^®^ (Sarchimor)	Starmaya F1 hybrid (CIR-SM01 × Marsellesa^®^)
2015–2016	30.1^b^	44.5^a^
2016–2017	31.1^b^	40.4^a^
2017–2018	30.1^b^	38.4^a^
Average	30.4 ± 0.6^b^	41.1 ± 3.1^a^

The productivity (measured in the number of bags of 46-kg green coffee beans per hectare) was evaluated during 3 years. Values followed by different letters are significantly different at P ≤ 0.05 (Fischer test of least significance difference). The comparison was based on an analysis of variance based on data collected on five blocks of 1 ha each.

### Evaluation of Off-Types for the Tree Phenotype and Leaf Rust Resistance of Starmaya F1 Hybrids

Phenotypic observations led to the identification of mean values of 3 and 16% of off-type plants in the fields of Marsellesa^®^ and Starmaya, respectively (**Table 6**). These off-type plants were characterized by tall plants, brown young leaves, and rust susceptibility. These unexpected phenotypes were probably a consequence of pollen contamination by wind and/or insects. The significant percentage of off-type plants observed in Starmaya fields could also be explained by the fact that one of the two parents (or both) was not sufficiently genetically fixed, therefore, still containing residual heterozygosity. Consequently, the cross between the two parents gave rise to a heterogeneous hybrid population instead of a homogeneous F1 progeny. Regarding leaf rust resistance, field observations identified 1 and 8% of plants susceptible to this disease in Marsellesa^®^ and Starmaya, respectively. As Marsellesa^®^ is a Sarchimor, coming from a cross between “Hibrido de Timor CIFC 832/2” and Villa Sarchi CIFC 971/10, it carried at least seven dominant resistance genes (*SH1*, *SH2*, *SH4*, *SH5*, *SH6*, *SH7*, and *SH9*) conferring a large spectrum of resistance to leaf rust (Eskes, 1989). The 1% of susceptible plants observed in the Marsellesa^®^ field argues in favor of this cultivar not being fully fixed. On the other hand, the rust susceptibility still observed in the Starmaya F1 hybrid probably came from an illegitimate offspring resulting from alien pollination or from residual Marsellesa^®^ heterozygosity.

**Table 6:** Evaluation of off-type frequencies based on plant phenotype and leaf rust sporulation for Marsellesa^®^ and Starmaya.

Plant phenotype	Without rust sporulation		With rust sporulation	
	Marsellesa^®^	Starmaya	Marsellesa^®^	Starmaya
Dwarf and green young leaves (normal)	294	254	2	23
Dwarf and brown young leaves (off-type)	6	25	0	0
Tall and green young leaves (off-type)	4	17	1	1
Tall and brown young leaves (off-type)	0	4	0	0
Total number of plants	300	300	3	24
Off-type frequency (%)	3	16	1	8

Evaluations were performed in 2016, on 300 plants from each cultivar 4 years after field planting (2012). Rust resistance incidence was assessed during the rainy season. Expected phenotypes correspond to normal plants (dwarf with green young leaves). Other phenotypes are considered as off-types.

Whatever the case, the fact that it was still possible to identify 16% of off-types and 8% of leaf rust-susceptible plants in the Starmaya F1 hybrid population is a disadvantage of hybrids propagated from seeds when compared with clonal F1 hybrids. Although off-types exist in F1 hybrid clones propagated by SE and are the result of somaclonal variation (aneuploidy), we have shown that a series of measures in the laboratory and nursery drastically limits their incidence of occurrence in the field (<1%) (Bobadilla Landey et al., 2013).

### Bean Quality and Cup Quality

The green bean size for Marsellesa^®^ and Starmaya was also investigated (**Table 7**). This analysis revealed that bean size distribution increased significantly in Starmaya compared with Marsellesa^®^. For example, 25% of Starmaya green beans were retained by sieve 19, while the percentage was only 2.5 and 5.8 for Marsellesa^®^ and Caturra red, respectively. In addition, 70% of the total green beans from Caturra red and Marsellesa^®^ were retained by sieves 16 to 18, while the same percentage was retained by sieves 17 to 19 for Starmaya. With 98% of green exportable coffee, the gain for Starmaya in terms of exportable green beans was thus seven points better than Caturra red but quite similar to that of Marsellesa^®^. In the world, there is considerable economic interest in producing the highest possible proportion of large size exportable beans (Vaast et al., 2006).

**Table 7:** Size distribution (expressed in percentage) of green coffee beans of Catura red, Marsellesa^®^, and Starmaya varieties of *C. Arabica* (2015–2016 harvest).

Sieve number	Bean size distribution by size (%)		
	Caturra red (control)	Marsellesa^®^	Starmaya F1 hybrid
n° 20 (big)	1.05	0.48	10.62
n° 19	5.74	2.48	25.05
n° 18	21.89	15.80	30.65
n° 17	33.86	38.45	16.91
n° 16 (medium)	14.46	22.91	8.02
n° 15	9.83	13.37	5.22
n° 14	3.80	4.62	1.11
n° 13 (small)	1.43	1.02	0.91
Defects	7.94	0.87	1.51
Total	100.00	100.00	100.00
Exportable (%)*	90.63	98.11	97.58

(*) Exportable = Total (100) – (defect + Sieve n°13).

In terms of cup quality, the results presented in **Table 8** show that the highest merit for aroma was obtained by Marsellesa^®^ beans. Beverages prepared with Starmaya and Marsellesa^®^ displayed the same slight fragrance of caramel, whereas Caturra red was characterized by a chocolate note. Marsellesa^®^ obtained better scores for acidity than the other Arabica samples. The same trend was observed for the overall flavor and aftertaste scores. The Starmaya beverage also had more body than Catura red and Marsellesa^®^ and more sweetness than Marsellesa^®^. Regarding the merit score, Marsellesa^®^ obtained the highest value (83.13), corresponding to very good scores for coffee beans usually produced at this altitude in Nicaragua (i.e., 950 m above sea level), while Starmaya and Caturra red obtained 82.5 and 82.0, respectively.

**Table 8:** Cup sensorial attributes of coffee beans from Caturra red, Marsellesa^®^, and Starmaya F1 hybrid of *C. arabica*.

Sensorial attributes	Caturra red (control)	Marsellesa^®^	Starmaya F1 hybrid
Quality	SHB	SHB^+^	SHB^+^
Aroma	7.63	7.75	7.63
Flavor	7.63	7.88	7.63
After taste	7.5	7.75	7.63
Acidity	7.5	8	7.75
Body	7.63	7.63	7.75
Uniformity	10	10	10
Clean cup	10	10	10
Balance	7.63	7.75	7.63
Sweetness	8.88	8.75	8.88
General impression	7.63	7.63	7.63
Final merite note	82	83.13	82.5

Sensorial attibutes were determined for the Caturra red cultivar corresponding to the control, for the parent Marsellesa*^®^* and for the Starmaya F1 hybrid of C. arabica, SHB, strictly hard bean.

#### SSR Analysis of the Hybrid and Its Progenitors

In order to understand the frequency of off-types observed in Marsellesa^®^ and Starmaya, a genotypic analysis was performed with SSR markers developed in earlier studies. Ten plants of Marsellesa^®^, of the sterile male CIR-SM01, and of the resulting F1 hybrid Starmaya were analyzed by SSR (**Table 9**). For Marsellesa^®^, all the tested plants showed the same allelic patterns for marker numbers Sat 11, Sat 225, Sat 235, Sat 24, and Sat 254, which scored with similar alleles. Two alleles were observed for markers Sat 29, Sat 32, Sat 47, and Sat 207. Taking into account that *C. arabica* is an allotetraploid (2n = 4x = 44) arising from natural hybridization between *C. canephora* and *C. eugenioides* (Lashermes et al., 1999), the observation of two bands was explained by the presence of two different loci in each sub-genome and two identical alleles for each locus for these markers. Under our study conditions, all the Marsellesa^®^ plants had the same allelic pattern, which tended to prove its high homozygosity. However, the presence of residual Marsellesa^®^ heterozygosity could not be ruled out, due to the limited number of SSR markers used (only 10 SSR markers).

**Table 9:** Simple sequence repeat allelic patterns observed for the two parents (Marsellesa^®^ and sterile male CIR-SM01) of the F1 hybrid Starmaya.

Primer name	Simple sequence repeat allelic patterns	
	**Marsellesa** **^®^**	**CIR-SM01**
Sat 11	A1A1 *(143)*	A2A2 *(145)*
Sat 225	B1B1 *(298)*	B2B3 *(269/296)*
Sat 24	C1C1 *(161)*	C2C3 *(153/172)*
Sat 32	D1D2 *(105/129)*	D1D3D4 *(105/121/133)*
Sat 47	E1E2 *(117/150)*	E1E3 *(117/137)*
Sat 235	F1F1 *(227)*	F2F3 *(259)*
Sat 254	G1G1 *(202)*	G2G2 *(220)*
Sat 29	H1H2 *(119/133)*	H1H3 *(119/135)*
Sat 244	I1I2 *(278/306)*	I1I3 *(278/302)*
Sat 207	J1J2 *(83/89)*	J1J3 *(83/93)*

This analysis was performed using 10 plants for each genotype. Values indicated in italics correspond to the length (in base pairs) of fragments amplified by polymerase chain reaction.

As regard the sterile male CIR-SM01, and for all SSR markers tested, all the plants presented the same patterns. This result was expected because all these plants were clonally propagated by SE. Markers Sat 11 and Sat 254 scored with similar alleles (homozygosity), while clearly residual heterozygosity was observed for marker loci Sat 32, Sat 24, and Sat 225, which identified the presence of three different alleles. We concluded that CIR-SM01 displayed constitutive heterozygosity that cannot be modified even when propagating the clone by SE.

The Starmaya F1 hybrid was assumed to be heterozygous because it received one set of chromosomes transmitted by the pollen of Marsellesa^®^ and the ovule of CIR-SM01. In fact, different patterns were observed for Starmaya (**Table 10**). For example, two different patterns were detected with markers Sat 24 and Sat 32 for plants P8 and P9 (C1C3/C1C2C3 and D1D2D4/D1D2D3, respectively) confirming that these markers were heterozygous in the sterile male parent.

**Table 10:** Allelic distribution patterns observed in 10 plants of Starmaya F1 hybrid. The 10 plants of Starmaya are numbered P1 to P10.

Simple sequence repeat primer	Allelic distribution patterns of the Starmaya F1hybrid									
	P1	P2	P3	P4	P5	P6	P7	P8	P9	P10
Sat 11	A1A2	A1A2	A1A2	A1A2	A1A2	A1A2	A1A2	A1A2	A1A2	A1A2
Sat 225	B1B2B3	B1B2B3	B1B2B3	B1B2B3	B1B2B3	B1B2b3	B1B2B3	B1B2B3	B1B2B3	B1B2B3
Sat 24	C2C3C4*	C1C3	C1C3	C1C3	C1C3	C1C3	C1C3	C1C3	C1C2C3	C1C3
Sat 32	D1D3D5**	D1D2D3	D1D2D3	D1D2D4	D1D2D3	D1D2D3	D1D2D3	D1D2D3	D1D2D4	D1D2D3
Sat 47	E1E2E3	E1E2E3	E1E2E3	E1E2E3	E1E2E3	E1E2E3	E1E2E3	E1E2E3	E1E2E3	E1E2E3
Sat 235	F1F2	F1F2	F1F2	F1F2	F1F2	F1F2	F1F2	F1F2	F1F2	F1F2
Sat 254	G1G2	G1G2	G1G2	G1G2	G1G2	G1G2	G1G2	G1G2	G1G2	G1G2
Sat 29	H1H2H3	H1H2H3	H1H2H3	H1H2H3	H1H2H3	H1H2H3	H1H2H3	H1H2H3	H1H2H3	H1H2H3
Sat 244	I1I2I3	I1I2I3	I1I2I3	I1I2I3	I1I2I3	I1I2I3	I1I2I3	I1I2I3	I1I2I3	I1I2I3
Sat 207	J1J2J3	J1J2J3	J1J2J3	J1J2J3	J1J2J3	J1J2J3	J1J2J3	J1J2J3	J1J23	J1J2J3

*C4 allele with a fragment length of 163 bp. **C5 allele with a fragment length of 111 bp.

The simple sequence repeat markers correspond to those described in **Table 2**. The results obtained for the 10 plants of F1 hybrid Starmaya were compared with those previously obtained for the two parents (Marsellesa*^®^* and Sterile Male CIR-SM01) and described in **Table 9**. The black codes identified in P1 plant indicate a pollen contamination. The gray codes identified in plants P8 and P9 indicate the presence of heterozygous alleles in the CIR-SM01 parent.

For the Starmaya P1 plant, two different alleles from both parents (alleles C4 and D5, respectively) were observed with marker loci Sat 24 and Sat 32. This result clearly indicates that this plant came from a cross-pollination involving alien pollen (i.e., not from Marsellesa^®^).

On the other hand, no Starmaya F1 hybrid plants had SSR patterns identical to the sterile male CIR-SM01, indicating that “sterile male × sterile male” crosses did not occur, thereby confirming the strict sterile male character of this accession. To reduce the residual heterozygosity carried by the pollen of Marsellesa^®^, we decided in 2017 to create new seed gardens using cultivars in generation F10. On the other hand, to avoid cross-pollination, we used windbreak hedges around the seed garden plot and protected the plants with an insect-proof net. Our current objective is to produce around one ton of Starmaya seeds in Nicaragua and Costa Rica in 2019, with a maximum of 5% off-types, which will be commercially acceptable.

## Conclusion

We demonstrated that Starmaya F1 hybrids had better agronomic characteristics in terms of vigor, bean size, and yield than the Marsellesa^®^ cultivar used as a parent or the Caturra red control. The Starmaya F1 hybrids also had a good cup quality compared with other traditional cultivars, such as Marsellesa^®^ and Caturra red. The results presented here are a proof of concept that it is possible to propagate a *C. arabica* F1 hybrid on a large scale from seeds, using the CIR-SM01 sterile male parent, by setting up seed gardens. The Starmaya cultivar is already included in the global catalog of the World Coffee Research foundation (https://varieties.worldcoffeeresearch.org/). The first commercial seeds (one ton) will be available in 2019. We believe that F1 hybrids produced using male sterility can produce low-cost seeds that will be readily available wherever Arabica is grown and that this innovation will reshape the coffee seed sector. Starmaya was recently recognized by the whole coffee industry as a promising cultivar representing a major breakthrough in the widespread production of F1 hybrids for farmers around the world (https://dailycoffeenews.com/2017/03/13/new-starmaya-hybrid-could-reshape-the-industry-says-world-coffee-research/). Under the BREEDCAFS H2020 European project (http://www.breedcafs.eu/), Starmaya has been introduced in Vietnam and Cameroon, where it is being compared with local varieties. On the other hand, new hybrids with the CIR-SM01 sterile male parent have been created and are under study.

The number of F1 hybrid varieties that can be created by seed is limited by the number of sterile male plants that can be found in coffee collections of wild trees collected in the species’ center of origin in Ethiopia. A consortium (World Coffee Research–CIRAD–Nestlé) is trying to identify male-sterility markers to create conventional sterile male cultivars. This would definitively open up the universe of known Arabica cultivars for use in breeding new F1 hybrids.

## Author Contributions

FG: corresponding author and the writer of the paper. LM: PHD student who statistically analyzed the results of the multilocal Starmaya trials in Nicaragua. EA: director of ECOM company in Nicaragua and facilitated the setup in the field of the experiment. PC: engineer in charge of producing planting material for the experiment in ECOM company (Nicaragua). MB: director of R&D in Nicafrance Fondation. JH: technician in charge of the harvest of the parcels in Nicafrance Fondation. PM: helped to revise the document. J-CB: helped to revise the document and to interpret the SSR results. ED: produced the MS plants by Somatic embryogenesis in the lab for the experiment. CP: general director of Nicafrance Fondation who provided the field in Cumplida farm to establish the Starmaya garden seed. HE: director of Coffee adapt team and cowriter of this paper. BB: breeder who created the Starmaya Hybrid and cowriter of this paper.

## Conflict of Interest

The authors declare that the research was conducted in the absence of any commercial or financial relationships that could be construed as a potential conflict of interest.
